# Complete plastome sequence of *Manilkara zapota* (L.) P.Royen (Sapotaceae)

**DOI:** 10.1080/23802359.2019.1667915

**Published:** 2019-09-19

**Authors:** Shu-Xiang Li, Xiao-Feng Zhang, Hong-Xin Wang, Zhi-Xin Zhu, Hua-Feng Wang

**Affiliations:** Hainan Key Laboratory for Sustainable Utilization of Tropical Bioresources, School of Life and Pharmaceutical Sciences, Hainan University, Haikou, China

**Keywords:** *Manilkara zapota*, plastome, phylogeny, genome structure, Sapotaceae

## Abstract

iThe plastome of *Manilkara zapota* is found to be 158,386 bp long with the typical quadripartite structure of angiosperms, contains two inverted repeats (IRs) of 26,099 bp each, a large single-copy (LSC) region of 87,745 bp, and a small single-copy (SSC) region of 18,443 bp. The plastome contains 114 genes, consisting of 80 unique protein-coding genes, 30 unique tRNA genes, and 4 unique rRNA genes. The overall A/T content in the plastome of M. zapota is 63.00%. The phylogenetic analysis indicated that *M. zapota* is close to *Sideroxylon wightianum* within Sapotaceae in this study.

## Introduction

*Manilkara zapota* (L.) P.Royen (Sapotaceae, Ericales) is a kind of arbor. It is 15–20 m tall. It originates in tropical America, cultivated in Guangdong, Guangxi, and Yunnan (Xishuangbanna) of China. Its fruit is edible, sweet, and delicious, its trunk milk is raw material of chewing gum, its seed contains 20% oil and its bark contains alkaloids, which can cure fever (Editorial Committee of Flora of China, Chinese Academy of Sciences 1987). Consequently, the genetic and genomic information is urgently needed to promote its systematic research and the development of its conservation value. Here, we report and characterize the complete plastome of *M. zapota* (GenBank accession number: MN295595). This is the first report of a complete plastome for *M. zapota.*

In this study, *M. zapota* was sampled from the greenhouse within Hainan University campus, Haikou, Hainan, China (110.327°E, 20.059°N). A voucher specimen (Wang et al., B254) was deposited in the Herbarium of the Institute of Tropical Agriculture and Forestry (HUTB), Hainan University, Haikou, China.

Around 6 Gb clean data were assembled against the plastome of *Camellia huana* (KY626040.1) (Wang et al. 2017) using MITObim v1.8 (Hahn et al. 2013). The plastome was annotated using Geneious R8.0.2 (Biomatters Ltd., Auckland, New Zealand) against the plastome of *C. huana* (KY626040.1). The annotation was corrected with DOGMA (Wyman et al. 2004).

The plastome of *M. zapota* was found to be 158,386 bp long with the typical quadripartite structure of angiosperms, containing two inverted repeats (IRs) of 26,099 bp, a large single-copy (LSC) region of 87,745 bp, and a small single-copy (SSC) region of 18,443 bp. The plastome contains 114 genes, consisting of 80 unique protein-coding genes, 30 unique tRNA genes, and 4 unique rRNA genes. The overall A/T content in the plastome of *M. zapota* is 63.00%, with the corresponding values of the LSC, SSC, and IR regions equalling 65.10, 69.80, and 57.10%, respectively.

**Figure 1.**
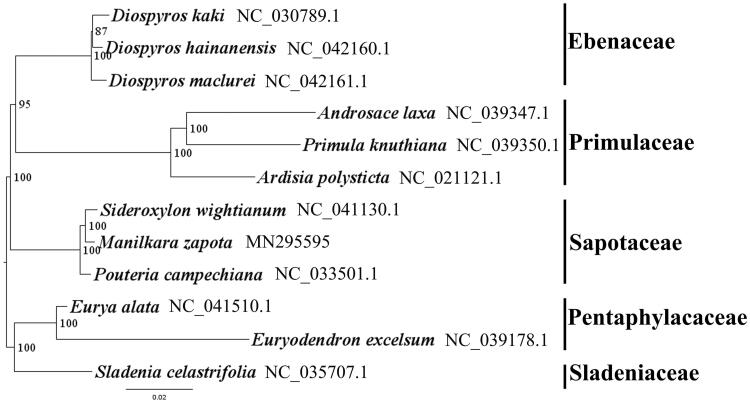
The best ML phylogeny recovered from 12 complete plastome sequences by RAxML. Accession numbers: *Manilkara zapota* MN295595, *Pouteria campechiana* NC_033501.1, *Sideroxylon wightianum* NC_041130.1, *Primula knuthiana* NC_039350.1, *Ardisia polysticta* NC_021121.1, *Androsace laxa* NC_039347.1, *Diospyros hainanensis* NC_042160.1, *Diospyros kaki* NC_030789.1, *Diospyros maclurei* NC_042161.1. Outgroups: *Eurya alata* NC_041510.1, *Euryodendron excelsum* NC_039178.1, *Sladenia celastrifolia* NC_035707.1.

We used RAxML (Stamatakis 2006) with 1000 bootstraps under the GTRGAMMAI substitution model to reconstruct a maximum-likelihood (ML) phylogeny of eight published complete plastomes of three families (Ebenaceae, Primulaceae and Sapotaceae), using 3 species from Pentaphylacaceae and Sladeniaceae as outgroups. The phylogenetic analysis indicated that *M. zapota* is close to *Sideroxylon wightianum* within Sapotaceae in this study (Figure 1). Most nodes in the plastome ML tree were strongly supported. The complete plastome sequence of *M. zapota* will provide a useful resource for the conservation genetics of this species as well as for the phylogenetic studies of Sapotaceae.
